# Supplementary material for: Iron alloys of volatile elements in the deep Earth’s interior
Source: Nat Commun. 2024 Apr 18;15:3320. doi: 10.1038/s41467-024-47663-0 (PMC11026407; doi:10.1038/s41467-024-47663-0)
Supplement: Supplementary file 1 — Supplementary Information [file 41467_2024_47663_MOESM1_ESM.pdf]

Supplementary Information for

"Iron Alloys of volatile elements in the Deep Earth's interior"

Yifan Tian[1,2], Peiyu Zhang[1,2], Wei Zhang[1,2,], Xiaolei Feng[3], Simon A. T. Redfern[4,3], Hanyu Liu[1,2,5,*]

[1]*Key Laboratory of Material Simulation Methods and Software of Ministry of Education, College of Physics, Jilin University, Changchun 130012, China*

[2]*State Key Laboratory of Superhard Materials, College of Physics, Jilin University, Changchun 130012, China*

[3]*School of Materials Science and Engineering, Nanyang Technological University, 50 Nanyang Avenue, Singapore, 639798 Singapore*

[4]*Asian School of the Environment, Nanyang Technological University, 50 Nanyang Avenue, Singapore, 639798 Singapore*

[5]*International Center of Future Science, Jilin University, Changchun 130012, China*

This Supplementary Information contains eleven Supplementary Figures, five Supplementary Tables and Supplementary References, summarized as follows.

-Supplementary Fig. 1 – Elemental abundance in the silicate Earth versus formation enthalpy of the Fe alloys with a substitution ratio of 1/128 ($Fe_{127}X$) at 20, 150 and 300 GPa.

-Supplementary Fig. 2 – Elemental abundance in the silicate Earth versus formation enthalpy of the Fe alloys with a substitution ratio of 1/54 ($Fe_{53}X$) and 1/128 ($Fe_{127}X$) at 20, 150 and 300 GPa.

-Supplementary Fig. 3 – Elemental abundance in the silicate Earth versus stabilities of the Fe alloys with a substitution ratio of 1/128 ($Fe_{127}X$) at 20, 150 and 300 GPa.

-Supplementary Fig. 4 – Comparison of primary wave velocities with different doping elements at 300 GPa.

-Supplementary Fig. 5 – Comparison of bulk sound velocities with different doping elements at 300 GPa.

-Supplementary Fig. 6 – Comparison of the Possion's ratios with different doping elements at 300 GPa.

-Supplementary Fig. 7 – Comparison of the shear moduli with different doping elements at 300 GPa.

-Supplementary Fig. 8 – Crystal structures of the simulation models.

-Supplementary Fig. 9 – Comparision of the calculated shear wave velocity (Vs) for *hcp*-Fe, $FeC_{0.0625}$, $FeO_{0.0625}$, $Fe_{0.9375}Si_{0.0625}$ at 0 K and high temperatures.

-Supplementary Fig. 10 – Mean square displacement (MSD) of Fe-As system.

-Supplementary Fig. 11 – Mean square displacement (MSD) of Fe-As and Fe-Se systems.

-Supplementary Table 1 – Classification of the elements.

-Supplementary Table 2 – Spin test calculations.

-Supplementary Table 3 – Formation enthalpies of the alloys.

-Supplementary Table 4 – Elastic properties of the alloys.

-Supplementary Table 5 – List of the elements and their atomic radii.

-Supplementary References

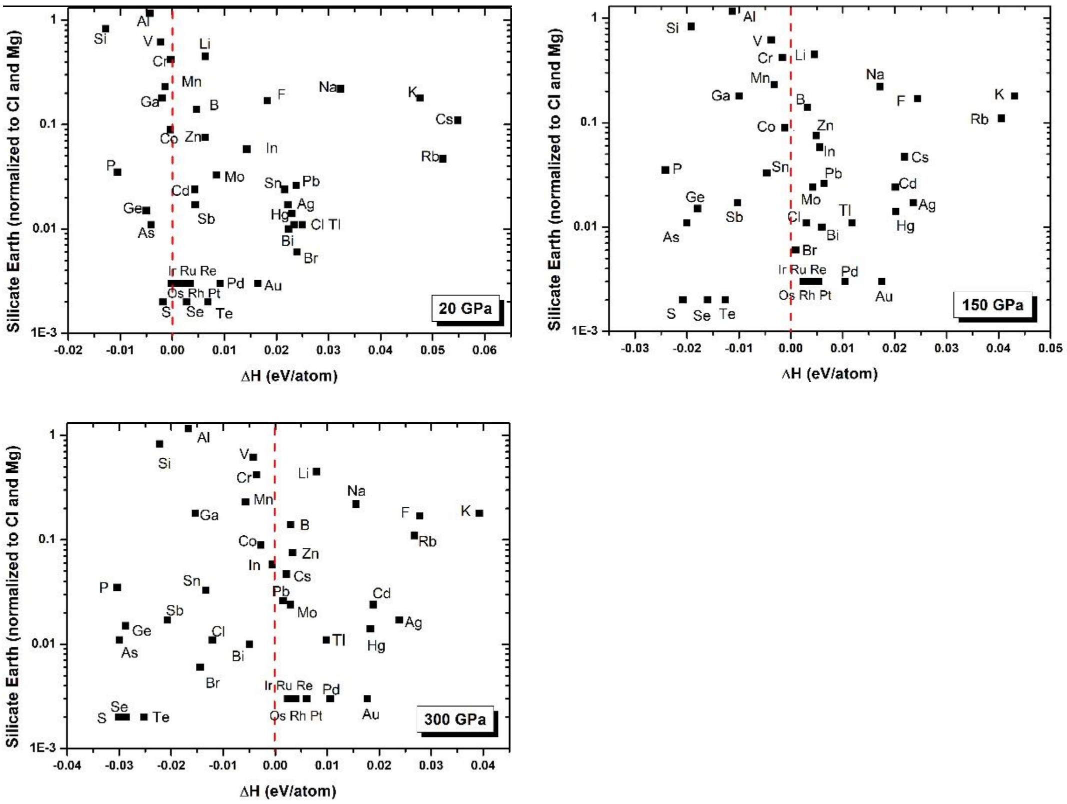

Figure S1. Elemental abundance in the silicate Earth versus formation enthalpy of the Fe alloys with a substitution ratio of 1/128 ($Fe_{127}X$) at 20, 150 and 300 GPa. Elemental abundances in the silicate Earth are ratioed to those in CI carbonaceous chondrites and normalized to $\frac{[Mg]_{Earth}}{[Mg]_{CI}} = 1.0$.

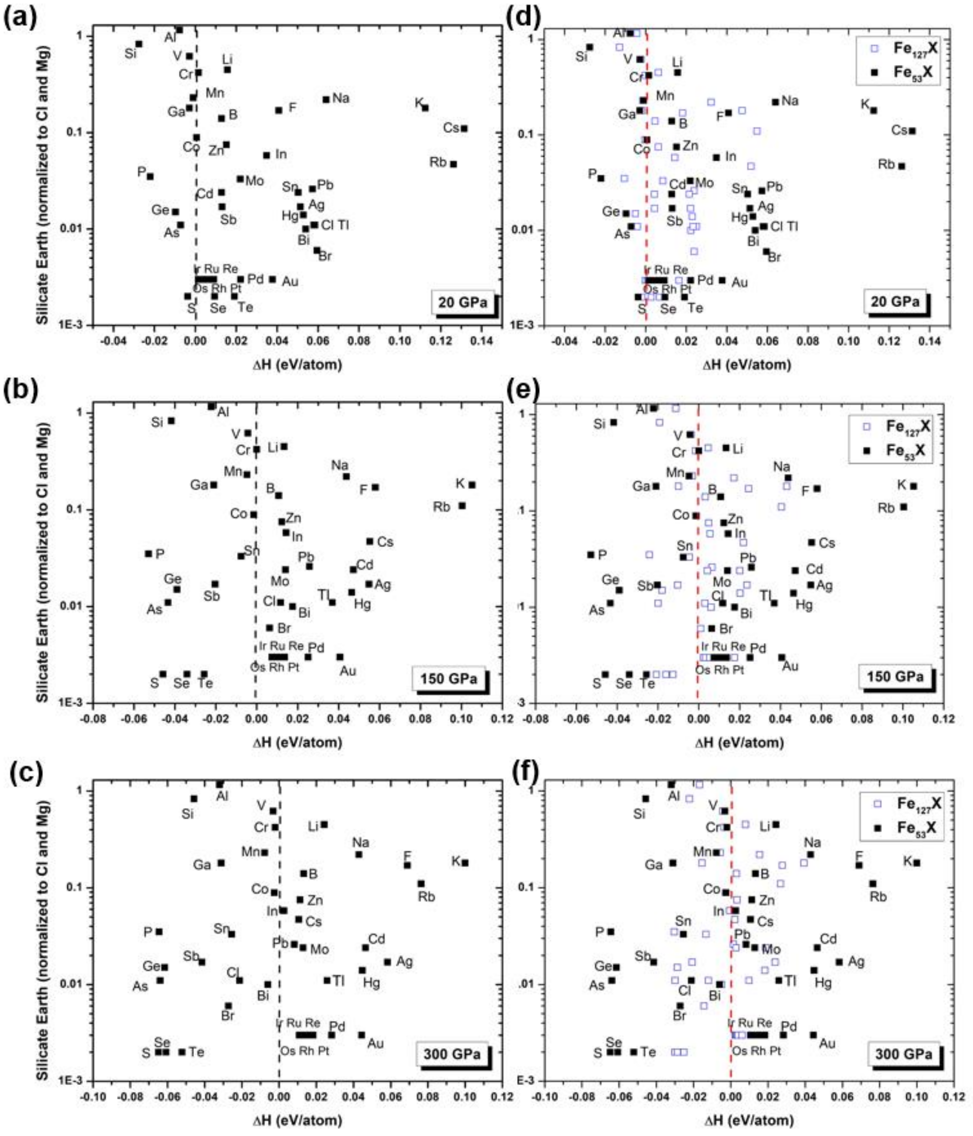

Figure S2. Elemental abundance in the silicate Earth versus formation enthalpy of the Fe alloys with a substitution ratio of 1/54 ($Fe_{53}X$) and 1/128 ($Fe_{127}X$) at 20, 150 and 300 GPa. (a), (b) and (c) represent substitution ratio of 1/54 at 20, 150 and 300 GPa, respectively. For easier comparison, the points from model $Fe_{127}X$ (in Figure S1) are plotted together with the points from model $Fe_{53}X$ on (d), (e) and (f) at 20, 150 and 300 GPa, respectively.

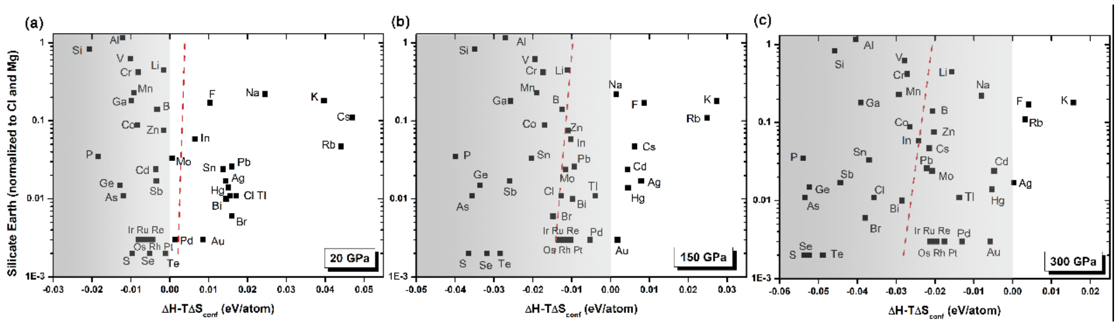

Figure S3. Elemental abundance in the silicate Earth versus stabilities of the Fe alloys with a substitution ratio of 1/128 ($Fe_{127}X$) at 20, 150 and 300 GPa. (a), (b) and (c) represent the results calculated at 20, 150 and 300 GPa, respectively. Elemental abundances in the silicate Earth are ratioed to those in CI carbonaceous chondrites and normalized to $\frac{[Mg]_{Earth}}{[Mg]_{CI}} = 1.0$. The horizontal axes are the terms of $\Delta H - T\Delta S_{conf}$ with different temperatures of 2000, 4000 and 6000 K at 20, 150 and 300 GPa, respectively. The red dashed lines represent the fitting of the data. Here, we focus on the slope of this fitting line: a positive slope indicates a positive correlation between the depletion of elements and the stability of the alloy, while a negative slope suggests the opposite. The slope of the fitting line is 6.5*10-4, 0.017 and 0.028 for 20, 150 and 300 GPa, respectively. Elements locate in shaded areas indicate that their alloys satisfy $\Delta H - T\Delta S_{conf} < 0$ under the corresponding conditions.

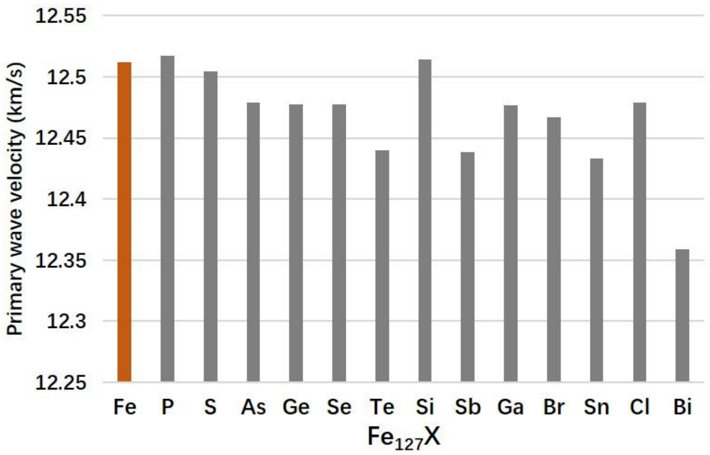

Figure S4. Comparison of primary wave velocities with different doping elements at 300 GPa. The substitutional ratio of these Fe alloys is 1/128 with a structure of Figure S1(a). For light elements, such as P and Si, would have an increasing effect on V_P of pure Fe. For other heavy elements, the V_P would be decreased after doping due to the increased density.

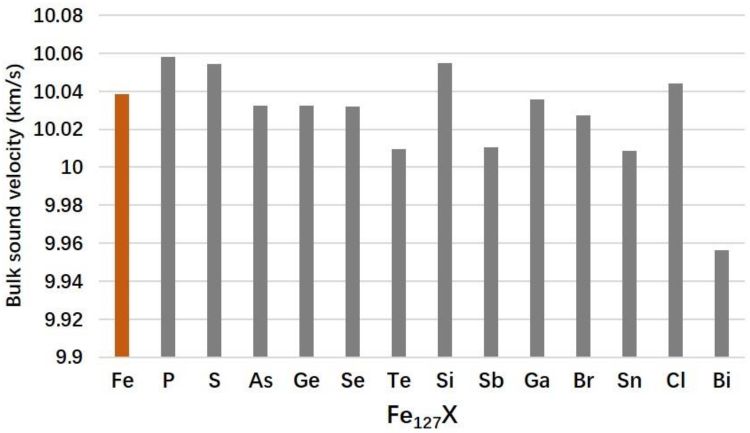

Figure S5. Comparison of bulk sound velocities with different doping elements at 300 GPa. The substitutional ratio of these Fe alloys is 1/128 with a structure of Figure S1(a).

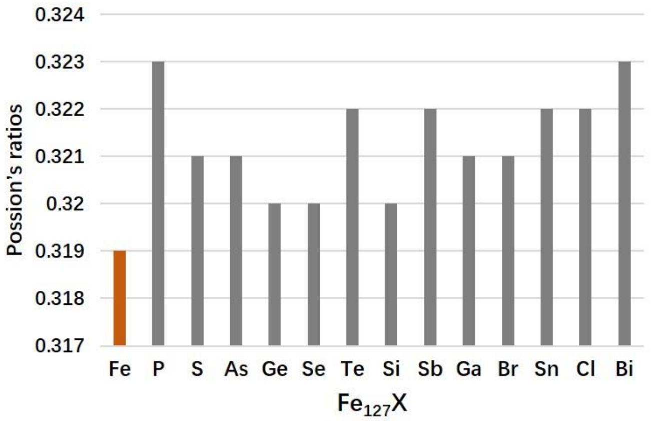

Figure S6. Comparison of the Possion's ratios with different doping elements at 300 GPa. The substitutional ratio of these Fe alloys is 1/128 with a structure of Figure S1(a).

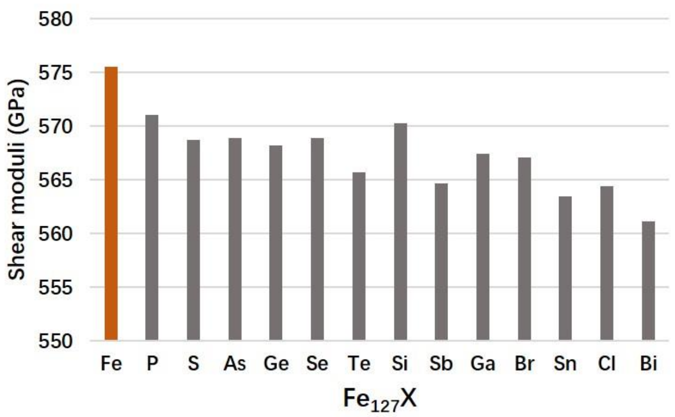

Figure S7. Comparison of the shear moduli with different doping elements at 300 GPa. The substitutional ratio of these Fe alloys is 1/128 with a structure of Figure S1(a).

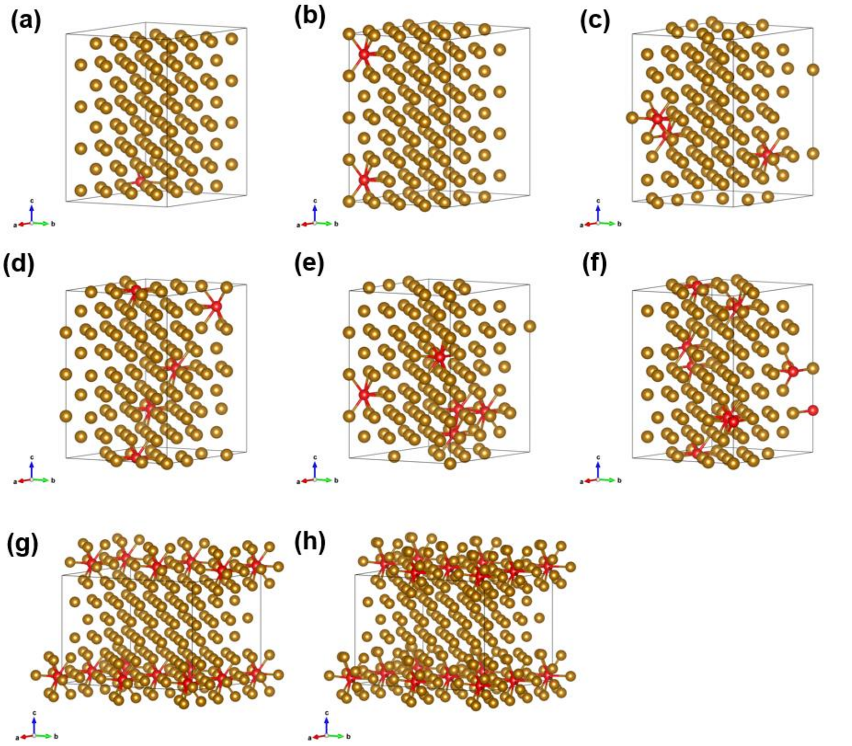

Figure S8. Crystal structures of the simulation models. The structures of the simulation models $Fe_{128-n}X_n$ (n=1, 2, 3, 4, 5 and 6, for a, b, c, d, e and f, respectively). Red and brown spheres represent impurity and Fe atoms, respectively. These structures are generated by special quasi-random structure (SQS) technique[1,2]. In these alloys, impurities atoms substitute Fe atoms randomly. (g) and (h) represent the models of $Fe_{140}X_4$ and $Fe_{144}X_4$, respectively. For each kind of impurity atom, the structure geometry optimizations based on the framework of density functional theory (DFT) would be generated independently.

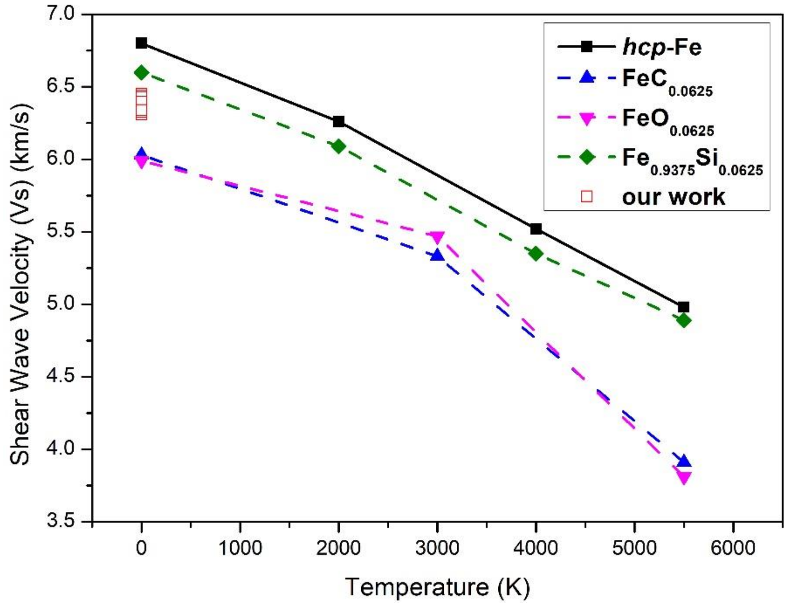

Figure S9. Comparision of the calculated shear wave velocity (*V*s) for *hcp*-Fe, $FeC_{0.0625}$, $FeO_{0.0625}$, $Fe_{0.9375}Si_{0.0625}$ at 0 K and high temperatures. Black solid line represents pure Fe, dash lines represent Fe alloys in previous works and red open squares represent the alloys in Figure 4b.

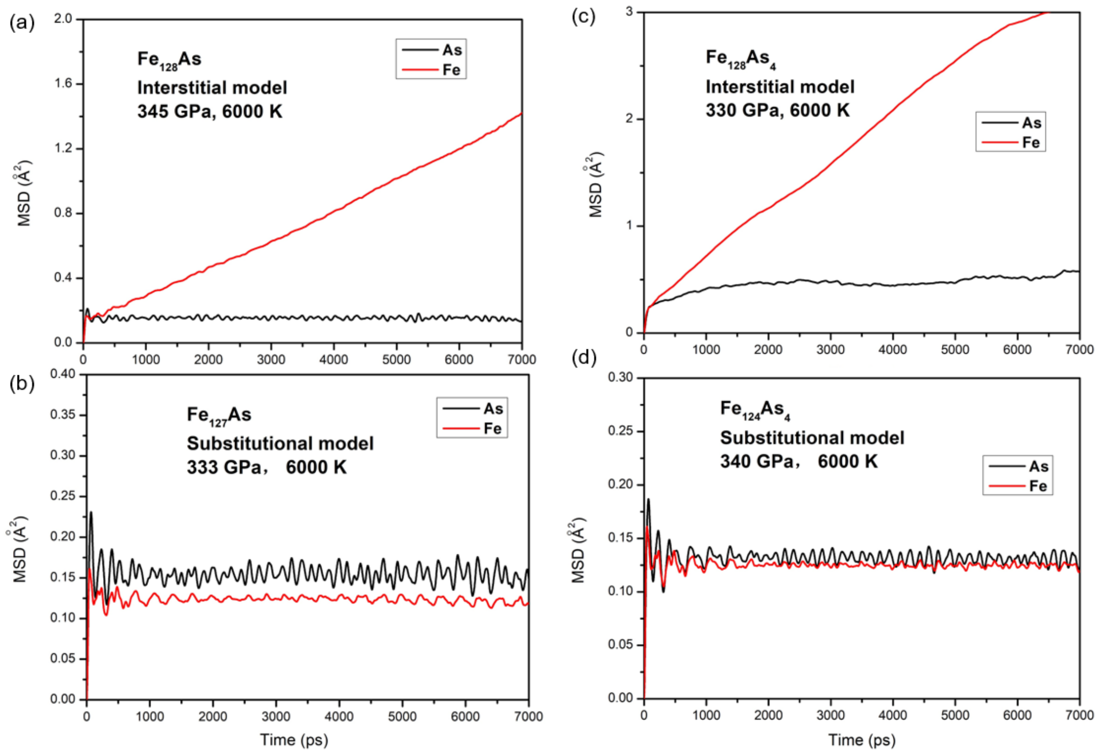

Figure S10. Mean square displacement (MSD) of Fe-As system. As and Fe in (a) $Fe_{128}As_1$, (b) $Fe_{127}As_1$, (c) $Fe_{128}As_4$ and (d) $Fe_{124}As_4$ represent interstitial and substitutional models with different ratios.

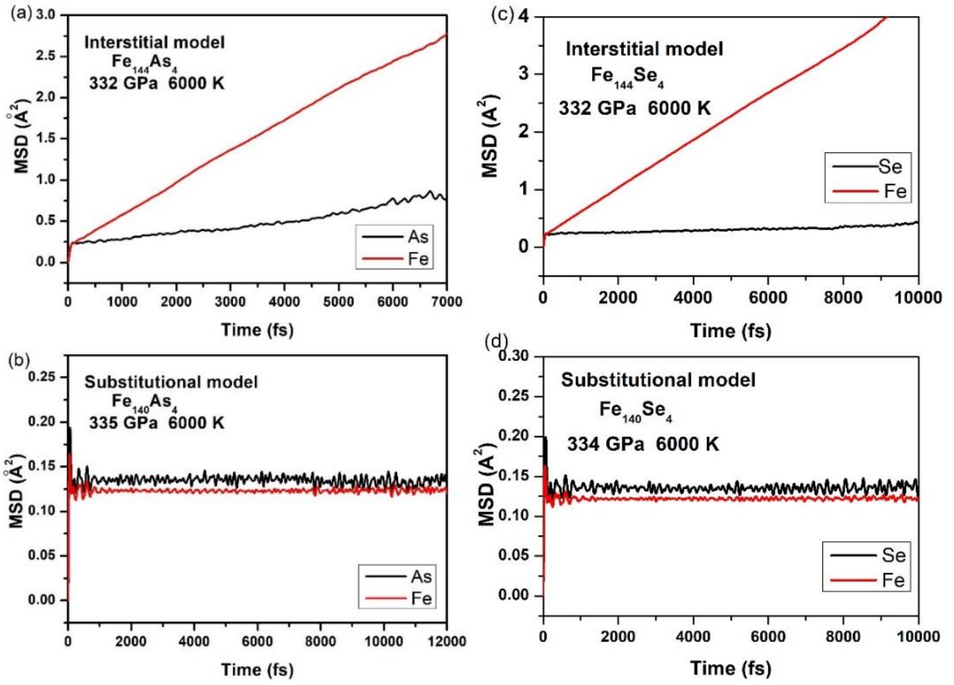

Figure S11. Mean square displacement (MSD) of Fe-As and Fe-Se systems. We have also performed addition simulations to randomly distribute the doping elements within the simulated cell, where we create a 2×2×1 supercell of 36 atoms (the simulated cell contains 144 atoms) and each a supercell of 36 atoms contains a doping element. Mean square displacement (MSD) of X and Fe in $Fe_{144}X_4$ and $Fe_{140}X_4$ at ~330 GPa and 6000 K could represent interstitial and substitutional models respectively. MSDs of (a), As and Fe in $Fe_{144}As_4$; (b), As and Fe in $Fe_{140}As_4$; (c), Se and Fe in $Fe_{144}Se_4$ and (d), Se and Fe in $Fe_{140}Se_4$. The MSDs of As and Se and Fe increase obviously with simulation time indicating a liquid state for the interstitial model, while the MSDs of the substitutional model show a solid state under Earth's inner core condition.

Table S1. **Classification of the elements**. The elements that we have considered in both main text and supplementary information are all included. Volatility of elements is classified according to 50% condensation temperatures (T_c) at 10^{-4} bar. In the current manuscript, we more focused on lithophile and chalcophile elements (non-siderophile elements as noted in the text) with moderate and high volatility, which are marked in green.

	**Lithophile**	**Siderophile**	**Chalcophile**
**Refractory** ($T_c \geqslant$ 1400 K)	Al, V	Mo, Ru, Rh, W, Re, Os, Ir, Pt	
**Transitional** (~1350 K > T_c > ~1250 K)	Mg, Si, Cr	Fe, Co, Ni, Pd	
**Moderately volatile** (~1250 K > T_c > ~800 K)	Li, B, Na, K, Mn, Rb, Cs	P, Cu, Ga, Ge, As, Ag, Sb, Au	
**Highly volatile** (T_c < 800 K)	F, Cl, Br, Zn	Tl, Bi	S, Se, Cd, In, Sn, Te. Hg, Pb

Table S2. Spin test calculations. The calculated enthalpies of $Fe_{53}Si$ with and without spin at 20, 150 and 300 GPa.

	**20 GPa (eV/atom)**	**150 GPa (eV/atom)**	**300 GPa (eV/atom)**
**non-spin-polarized**	-6.9602	-0.0756	6.6445
**spin-polarized**	-6.9587	-0.0755	6.6445

Table S3. Formation enthalpies of the alloys. The calculated formation enthalpy (eV/atom) of the alloys with a substitution ratio of 1/128 ($Fe_{127}X$) at 20, 150 and 300 GPa.

	**ΔH @ 20 GPa**	**ΔH @ 150 GPa**	**ΔH @ 300 GPa**
**F**	0.0182	0.0243	0.0277
**S**	-0.0017	-0.0207	-0.030
**Si**	-0.01283	-0.0192	-0.0221
**Al**	-0.0043	-0.0112	-0.0167
**P**	-0.0105	-0.0241	-0.0303
**Ge**	-0.0050	-0.0179	-0.0287
**Ga**	-0.0019	-0.0099	-0.0154
**Sn**	0.0084	-0.0046	-0.0133
**Pb**	0.0238	0.0063	0.0014
**As**	-0.0041	-0.0199	-0.0299
**Sb**	0.0044	-0.0102	-0.0207
**Bi**	0.0223	0.0059	-0.0049
**Se**	0.0027	-0.0160	-0.0286
**In**	0.0143	0.0055	-0.000
**Tl**	0.0249	0.0118	0.009
**Br**	0.0238	0.0009	-0.0144
**Cl**	0.0234	0.0029	-0.0120
**Te**	0.0068	-0.0126	-0.0252
**Zn**	0.0062	0.0048	0.0033
**Na**	0.0323	0.0171	0.0155
**K**	0.0475	0.0431	0.0392
**Rb**	0.0548	0.0405	0.0267
**Mn**	-0.0013	-0.0032	-0.0056
**Li**	0.0063	0.0045	0.0079
**B**	0.0046	0.0031	0.0029
**V**	-0.0022	-0.0037	-0.0042
**Cr**	-0.0003	-0.0016	-0.0035
**Ag**	0.0222	0.0236	0.0238
**Cd**	0.0215	0.0201	0.0188
**Pd**	0.0092	0.0104	0.0105
**Co**	-0.0003	-0.0011	-0.0027
**Mo**	0.0043	0.0042	0.0028
**Au**	0.0164	0.0175	0.0177
**Ru**	0.0016	0.0027	0.0023
**Rh**	0.0027	0.0040	0.0040
**Re**	0.0013	0.0029	0.0028
**Ir**	-0.0001	0.0023	0.0032
**Os**	0.0008	0.0032	0.0039
**Pt**	0.0034	0.0053	0.0060

Table S4. Elastic properties of the alloys. Densities (ρ), elastic constants (Cij), moduli (B and G) and Poisson's ratio of the representative models at 300 GPa and 0K.

	ρ (g/cm³)	C_{11} (GPa)	C_{12} (GPa)	C_{13} (GPa)	C_{33} (GPa)	C_{44} (GPa)	C_{66} (GPa)	B (GPa)	G (GPa)	*v*
**Pure Fe (Fe_{128})**	**13.753**	**2169.3**	**1072.8**	**900.2**	**2388.0**	**522.9**	**547.2**	**1385.84**	**575.49**	**0.318**
$Fe_{127}P$	13.713	2163.7	1076.4	902.3	2394.9	515.9	542.8	1387.22	571.03	0.319
$Fe_{126}P_2$	13.667	2159.3	1078.5	901.2	2395.0	505.5	539.5	1386.12	565.65	0.320
$Fe_{125}P_3$	13.619	2148.0	1083.6	906.9	2383.9	499.2	532.6	1385.92	558.32	0.322
$Fe_{124}P_4$	13.576	2136.6	1087.4	911.2	2368.0	494.1	524.1	1384.56	550.52	0.324
$Fe_{123}P_5$	13.533	2129.1	1090.3	916.1	2355.6	486.8	514.0	1383.84	541.41	0.327
$Fe_{122}P_6$	13.488	2117.7	1095.6	919.7	2348.4	479.6	509.2	1383.24	536.63	0.328
$Fe_{127}Si$	13.700	2160.5	1077.3	899.6	2391.2	516.1	540.6	1385.10	570.27	0.319
$Fe_{126}Si_2$	13.644	2151.7	1081.9	897.8	2388.5	505.1	534.1	1383.00	563.18	0.321
$Fe_{124}Si_4$	13.532	2131.3	1087.8	901.9	2367.6	494.0	522.3	1379.24	550.66	0.324
$Fe_{123}Si_5$	13.476	2128.8	1086.2	898.8	2360.0	484.4	514.2	1377.33	545.60	0.325
$Fe_{127}As$	13.769	2158.7	1076.9	902.4	2390.7	514.3	540.3	1385.80	568.87	0.319
$Fe_{126}As_2$	13.783	2153.1	1079.2	903.2	2387.9	503.7	536.2	1384.97	562.58	0.321
$Fe_{125}As_3$	13.796	2142.9	1082.8	906.8	2376.3	496.5	531.4	1383.68	555.89	0.323
$Fe_{127}Ge$	13.765	2157.6	1078.7	901.7	2389.5	514.1	538.5	1385.49	568.19	0.320
$Fe_{126}Ge_2$	13.774	2146.3	1084.0	903.1	2381.9	501.2	530.5	1383.78	558.86	0.322
$Fe_{125}Ge_3$	13.783	2138.5	1085.7	904.7	2375.6	495.6	527.9	1382.40	554.07	0.323
$Fe_{127}S$	13.716	2159.9	1074.7	905.0	2390.6	513.5	541.6	1386.68	568.71	0.320
$Fe_{126}S_2$	13.679	2156.7	1076.1	906.2	2388.5	501.5	539.3	1386.52	562.63	0.321
$Fe_{124}S_4$	13.601	2129.3	1087.1	919.1	2360.0	488.5	519.4	1385.20	544.58	0.326
$Fe_{127}Se$	13.765	2159.8	1075.7	904.2	2390.4	514.1	541.1	1386.49	568.90	0.320
$Fe_{126}Se_2$	13.798	2155.4	1075.4	904.9	2387.4	504.1	539.3	1385.32	563.70	0.321
$Fe_{127}Te$	13.822	2153.1	1078.5	904.1	2385.5	511.7	536.6	1385.01	565.68	0.320
$Fe_{126}Te_2$	13.888	2142.5	1079.5	905.7	2377.1	501.8	531.1	1382.51	558.34	0.322
$Fe_{127}Sb$	13.777	2150.7	1079.6	902.9	2383.6	511.0	534.8	1384.01	564.66	0.320
$Fe_{127}Ga$	13.764	2156.8	1080.5	903.1	2387.9	514.1	537.2	1386.22	567.40	0.320
$Fe_{127}Br$	13.777	2155.8	1073.5	905.9	2384.4	512.5	540.2	1385.22	567.10	0.320
$Fe_{127}Sn$	13.807	2148.2	1080.5	902.6	2380.2	510.2	533.0	1383.13	563.41	0.321
$Fe_{127}Cl$	13.723	2151.7	1073.1	907.7	2378.9	509.6	538.4	1384.41	564.41	0.321
$Fe_{127}Bi$	13.955	2145.5	1080.9	905.3	2376.6	507.4	531.8	1383.36	561.08	0.321

Table S5. List of the elements and their atomic radii. Impurity elements and their atomic radii considered in this work are shown here.

Element	Atomic Radii (pm)	Element	Atomic Radii (pm)
F	42	K	243
S	88	Rb	265
Si	111	Mn	161
Al	118	Li	167
P	98	B	87
Ge	125	V	171
Ga	136	Cr	166
Sn	145	Ag	165
Pb	154	Cd	161
As	114	Pd	169
Sb	133	Co	152
Bi	143	Mo	190
Se	103	Au	174
In	156	Ru	178
Tl	156	Rh	173
Br	94	Re	188
Cl	79	Ir	180
Te	123	Os	185
Zn	142	Pt	177
Na	190	Fe	156

Reference:

[1] Alex Zunger, S.-H. Wei, L. G. Ferreira, and James E. Bernard, Special quasirandom structures, *Phys. Rev. Lett*. **65,** 353 (1990).
[2] S.-H. Wei, L. G. Ferreira, James E. Bernard, and Alex Zunger, Electronic properties of random alloys: Special quasirandom structures, *Phys. Rev. B* **42**, 9622 (1990).
